# Forecasting lung cancer incidence, mortality, and prevalence to year 2030

**DOI:** 10.1186/s12885-021-08696-6

**Published:** 2021-09-03

**Authors:** Erik Jakobsen, Karen Ege Olsen, Mette Bliddal, Malene Hornbak, Gitte F. Persson, Anders Green

**Affiliations:** 1 OPEN Open Patient data Explorative Network, Odense University Hospital and University of Southern Denmark, Odense, Denmark; 2 Danish Lung cancer Registry, Department of Thoracic Surgery, Odense University Hospital, DK-5000 Odense C, Denmark; 3 Department of Pathology, Odense University Hospital, Odense, Denmark; 4 Institute of Applied Economics and Health Research (ApHER), Copenhagen, Denmark; 5 AstraZeneca, Copenhagen, Denmark; 6 Department of Oncology, Herlev-Gentofte Hospital, Copenhagen, Denmark

**Keywords:** Lung cancer, Epidemiology, Incidence, Mortality, Prevalence forecasting

## Abstract

**Background:**

Lung cancer incidence and prevalence is increasing worldwide and there is a focus on prevention, early detection, and development of new treatments which will impact the epidemiological patterns of lung cancer. The clinical characteristics and the trends in incidence, mortality, and prevalence of lung cancer in Denmark from 2006 through 2015 are described and a model for predicting the future epidemiological profile of lung cancer through 2030 is introduced.

**Methods:**

The study population comprised all cases of lung cancer, registered in the Danish Cancer Registry, who were alive on January 1, 2006 or had a first-time ever diagnosis of lung cancer during 2006 through 2015. Information on morphology, stage of the disease, comorbidity and survival was obtained from other Danish health registers. Based on NORDCAN data and estimated patient mortality rates as well as prevalence proportions for the period 2006 through 2015, future case numbers of annual incidence, deaths, and resulting prevalence were projected.

**Results:**

A total of 44.291 patients were included in the study. A shift towards more patients diagnosed with lower stages and with adenocarcinoma was observed. The incidence increased and the patient mortality rate decreased significantly, with a doubling of the prevalence during the observation period. We project that the numbers of prevalent cases of lung cancer in Denmark most likely will increase from about 10,000 at the end of 2015 to about 23,000 at the end of 2030.

**Conclusions:**

Our findings support that lung cancer is being diagnosed at an earlier stage, that incidence will stop increasing, that mortality will decrease further, and that the prevalence will continue to increase substantially. Projections of cancer incidence, mortality, and prevalence are important for planning health services and should be updated at regular intervals.

## Introduction

Worldwide, the incidence of lung cancer has been described as an epidemic with variabilities across countries according to socio-economic, historical, and cultural characteristics [1]. This also applies to the Western European countries where different trends in incidence have been attributed to differences in smoking patterns and socio-economic prerequisites [2].

Lung cancer is associated with huge costs for patients and society, and there is an increasing focus on prevention, early detection with screening, and development of new treatments [3]. When introduced, such new modalities will impact the future epidemiological patterns of lung cancer. This dynamic senario calls for tools to monitor a comprehensive description of the current and future trends in the epidemiology of lung cancer.

In this paper we describe the clinical characteristics and the trends in incidence, mortality, and prevalence of lung cancer in Denmark from 2006 through 2015. Based on these observations we introduce a model for predicting the future epidemiological profile of lung cancer through 2030.

## Material and methods

### Data sources

The study population comprised all patients with lung cancer in Denmark from 2006 through 2015. The study is a part of the CEDAR Study (Cancer Impact in Denmark Study). The CEDAR Study is a nationwide, observational study on patient populations with cancer of the lung, breast, bladder, ovary, and prostate.

Patients have been ascertained from the Danish Cancer Registry [4], and by record linkage at person-level information from the Danish Civil Person Register [5], the Danish Cancer Registry, the Danish National Patient Register [6], the Danish Register of Causes of Death [7], and the Pathology Register [8], cancer trajectories have been established with profiles of the patients by stage, tumour morphology and comorbidity at diagnosis. Furthermore, milestones on the introduction of relevant new cancer treatments and other interventions were established.

### Study population and data

The study population comprised patients with a diagnosis of lung cancer registered in the Cancer Registry [ICD10 code C33-C34]. We included all prevalent patients resident in Denmark and alive on January 1, 2006, and all patients with a first-time ever diagnosis of lung cancer from January 1, 2006 through December 31, 2015.

For each case of cancer in the Danish Cancer Registry there is one tumour morphology SNOMED code recorded. The codes were grouped in accordance with the morphology classification in the Danish Lung cancer Register [9]. In the Danish Cancer Registry, cases of lung cancer are registered with codes in the TNM classification system for disease stage. These data were converted to disease stage based on TNM UICC version 7 [10]. Based on hospital diagnoses registered during the 10 years period prior to the diagnosis of lung cancer, we established a Charlson Comorbidity Index score [11] for each patient. Patients were followed based on data from the Danish Civil Registration System until death, emigration, or December 31, 2015, whichever came first. Annual population counts by sex and age were obtained from national census data at Statistic Denmark and used as denominator when calculating descriptive numbers.

### Methods of analysis: observation period 2000 through 2015

Incidence rates were estimated annually (per 10,000) as the number of new cases divided by the estimated person-time at risk in the background population and expressed per 10.000. The data were grouped by sex and age in the intervals 40–59, 60–69, 70–79, and > 80 years, respectively, thereby truncating the risk population at 40 years as the lower level. The very few cases of lung cancer with diagnosis below age 40 years were allocated to the age group 40–59 years. Prevalence proportions were estimated (per 10.000) as the number of patients alive divided by the total population size on an annual basis with reference to December 31 each year. Patient mortality rates were estimated annually (per 100 person-years at risk in the patient population) as the number of deaths (regardless of cause of death) divided by the estimated patient-time at risk. Poisson regression analysis (Stata version 15.0) was applied to assess potential determinants of the incidence and mortality rates. Chi-square analyses were used to test for trends in proportions.

### Methods of analysis: projecting future incidence, mortality and prevalence

The projection of the future numbers of annual incident cases, deaths, and resulting prevalent case numbers was made for the period 2016 through 2030 and was based on the principles in a ‘stock and flow’ model for a closed population as illustrated for diabetes with or without complications [12]. The principle is that the annual change in the prevalent case number is a result of the number of prevalent cases a year before plus the new cases minus the number of deaths in the patient population during the year concerned. These principles were implemented by taking the latest observed prevalence (here, by the end of 2015), add the projected incident cases for the next year (by the assumed incidence rate multiplied by the estimated population at risk) minus the projected number of deaths in the patient population (by the assumed mortality rate multiplied by the estimated number of patient-years at risk of death) to obtain the projected prevalence numbers for the following year, and so forth. The projections were stratified by sex and groups of age at diagnosis (40–59, 60–69, 70–79, > 80 years), and we used December 31st as the prevalence data for each year. Trends in incidence rates were based on data from NORDCAN, a database on cancer statistics and which offers aggregated data on cancer cases in the Nordic countries [13]. From the NORDCAN download resource we obtained numbers of incident cases, aggregated by sex and 5-years age classes of lung cancer data (ICD10 codes C33-C34) in Denmark on an annual basis for the years 1999 through 2015. After 2015 projected incident cases numbers projected by NORDCAN were available in categories aggregated by sex and 5-years calendar and age groups and were transformed to annual numbers using floating averaging over five calendar years. After further grouping to sex and age classes 40–59, 60–69, 70–79, and > 80 years as described above, observed and projected sex and age-specific incidence rates were obtained using national census data from Statistics Denmark, including demographical projections through 2031. Data on deaths and mortality rates of relevance for the present purpose are not available in the NORDCAN database. To fit the data for the observation period as best as possible, we used an exponential model (the GROWTH function in Excel) applied to the present observed mortality rates to predict the future rates. For reference purposes we have established the hypothetical scenario with all incidence and mortality rates kept constant at the levels of year 2015. For the projection analyses data were stratified for sex and age groups 40–59, 60–69, 70–79, and > 80 years. We quantified the individual components driving the change in incidence and prevalence over calendar time.

The basic epidemiological analyses were made in Excel.

### Ethical approval

#### Ethical approval statement

All experimental protocols were approved by the Danish Data Protection Agency under j.nr. 2008-58-0035.

#### Informed consent

Due to Danish legislation on register-based research, no further permissions were required, including informed consent, waived by the Danish Protection Agency.

#### Guidelines/accordance

All methods were carried out in accordance to relevant guidelines and regulations.

## Results

### Epidemiological characteristics 2000 through 2015

Over time, the incidence increased in case numbers, with a trend towards gender balance from a predominance of men. The prevalence doubled in the observed period, from 5.967 patients by end of year 2005 to 10.394 patients by the end of 2015. The annual number of deaths was higher for males than for females, with a stable total number around 4000 annually in spite of the increasing prevalence (Table 1).

**Table 1:** Epidemiological key numbers of lung cancer in Denmark 2006 through 2015

Year	New cases			Deaths			Prevalence (end of year)			Population size, aged > 40 years (end of year)		
	Males	Females	Total	Males	Females	Total	Males	Females	Total	Males	Females	Total
2005	–	–	–	–	–	–	3071	2896	5967	1,291,417	1,394,382	2,685,799
2006	2168	1929	4097	2158	1752	3910	3080	3073	6153	1,311,242	1,411,197	2,722,439
2007	2273	2018	4291	2065	1755	3820	3284	3334	6618	1,328,555	1,424,974	2,753,529
2008	2226	2021	4247	2118	1809	3927	3396	3546	6942	1,343,866	1,437,448	2,781,314
2009	2261	2044	4305	2119	1855	3974	3537	3736	7273	1,356,054	1,448,171	2,804,225
2010	2294	2218	4512	2133	1900	4033	3698	4052	7750	1,368,302	1,459,385	2,827,687
2011	2277	2214	4491	2068	1883	3951	3908	4385	8293	1,382,700	1,472,532	2,855,232
2012	2359	2182	4541	2140	1880	4020	4124	4687	8811	1,397,701	1,487,226	2,885,927
2013	2239	2302	4541	2100	1901	4001	4264	5089	9353	1,411,337	1,500,676	2,912,013
2014	2417	2247	4664	2193	1935	4128	4489	5400	9889	1,426,661	1,515,371	2,942,032
2015	2296	2306	4602	2140	1959	4099	4646	5748	10,394	1,437,966	1,527,815	2,965,781
All years	22,810	21,481	44,291	21,234	18,629	39,863	–	–	–			

### Stage, pathology and comorbidity

**Fig. 1:**
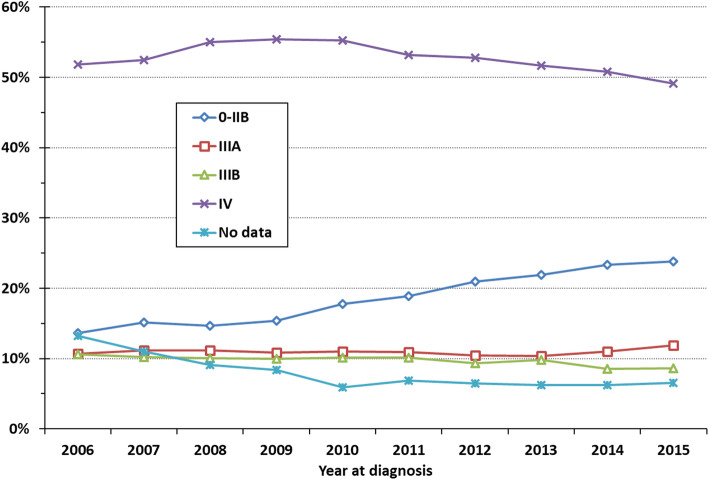
Trend in high-level stage distribution, LC patients diagnosed 2006–2015 (incl)

Of the 44,291 patients diagnosed from 2006 through 2015 a detailed TNM stage was not reported for 8%. The stage distribution is presented in (Fig. 1) where it is seen that the relative numbers of patients in stage classes IV and IIIB declined weakly whereas a relative increase in patients in stage classes 0 – IIIA, was observed. The increasing trend in the proportion of patients diagnosed with stage classes 0-IIIA was statistically significant (*P* < 0.0001).

**Fig. 2:**
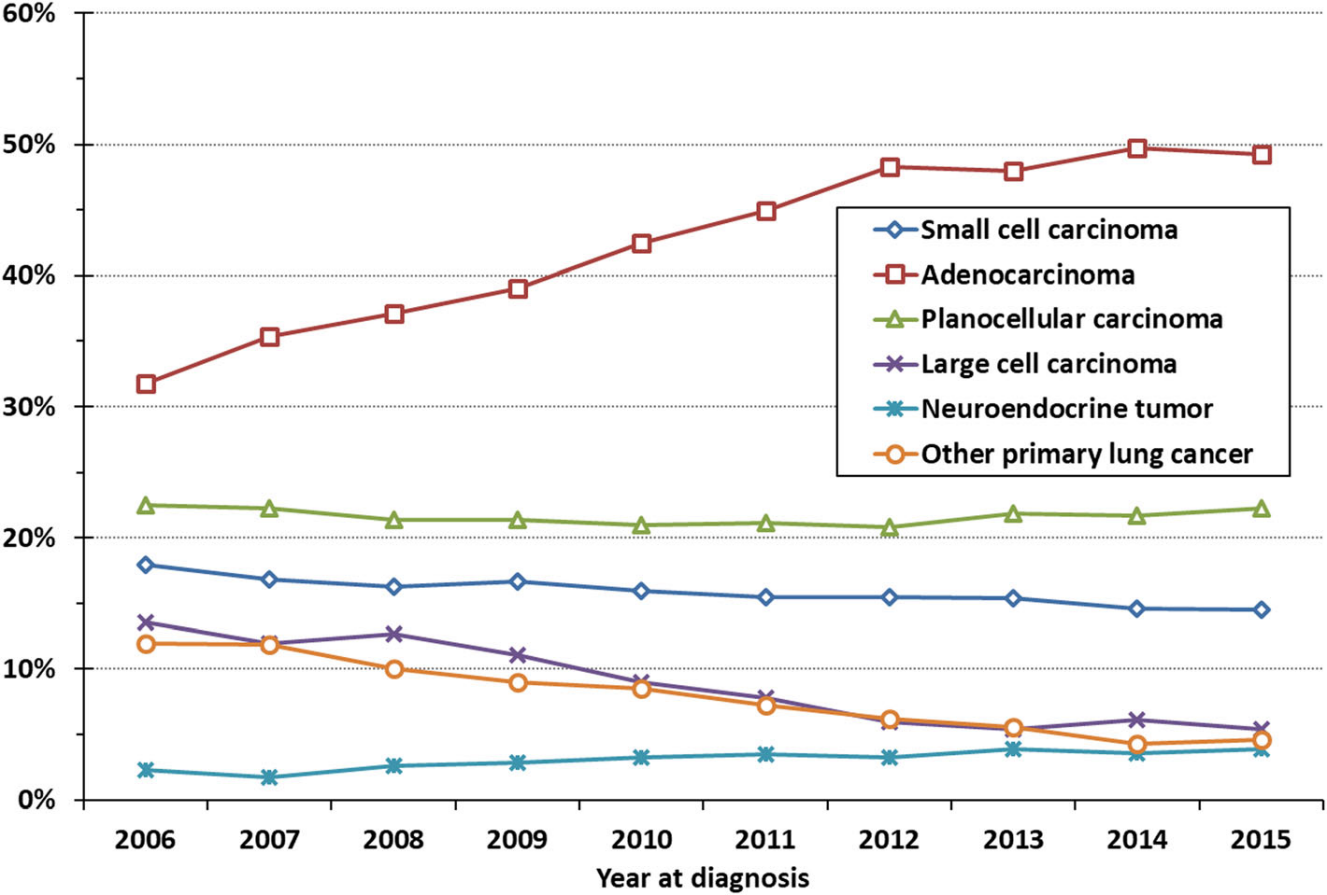
Trends in the distribution of high-level morphology categories, LC patients diagnosed 2006–2015 (incl.), with conclusive pathology (*N* = 40,457)

The number of patients without a specific morphology diagnosis decreased from 14% in 2006 to 8% in 2015. The proportion of patients diagnosed with adenocarcinoma increased statistically significant (*P* < 0.0001) with a corresponding decrease in the number of patients with large cell carcinoma and other primary lung cancer (Fig. 2). At high level morphology classification, the proportions of patients with squamous cell and small cell carcinomas have been relatively stable.

The proportion of patients with comorbidity (CCI score > 0) increased over time (Table 2). Thus, the proportion of patients without comorbidity (CCI score = 0) decreased from 64% in 2006 to 59% in 2015. This trend was statistically significant (*P* < 0.0001).

**Table 2:** Distribution of Charlson Comorbidity Index at diagnosis, LC patients diagnosed 2006–2015 (incl)

	Year at diagnosis										
	2006	2007	2008	2009	2010	2011	2012	2013	2014	2015	Total
CCI											Grand Total
0	64%	63%	63%	63%	62%	61%	62%	60%	60%	59%	27,292 (62%)
1	17%	17%	18%	17%	18%	18%	17%	18%	18%	17%	7799 (18%)
2	11%	11%	11%	12%	12%	12%	12%	13%	13%	13%	5348 (12%)
> 2	8%	8%	8%	8%	9%	9%	9%	9%	9%	10%	3852 (9%)

The statistical analysis of the incidence and mortality rates is summarized in (Table 3). During the study period, the incidence rate for females was 20% lower (*P* < 0.0001) compared with the rate for males. For both sexes, the incidence rates increased statistically significantly (*P* < 0.0001) for the age groups 60–69 and 70–79 years using the age group < 60 years as the reference. The incidence rate level for the age group > 80 years was interposed between those by the age groups 60–69 and 70–79 years. With the inclusion of age groups and sex as covariates the incidence rate decreased statistically significantly by 0.5% annually (*P* = 0.005).

**Table 3:** Statistical analysis of incidence and mortality

INCIDENCE			
Covariate	HR	95% C.I.	*P* value
Sex			
Male (ref.)	(1)	–	–
Females	0.803	0.789; 0.819	< 0.0001
Year (ref.:2006)	0.995	0.992; 0.999	0.005
AgeGrp			
< 60 (ref.)	(1)	–	–
60–69	4.499	4.374; 4.628	< 0.0001
70–79	8.286	8.059; 8.520	< 0.0001
> 80	6.977	6.755; 7.207	< 0.0001
MORTALITY			
Covariate	HR	95% C.I. (a)	*P* value
Sex			
Male (ref.)	(1)	–	–
Females	0.809	0.761; 0.859	< 0.0001
Year (ref.: 2006)	0.944	0.934; 0.954	< 0.0001
AgeGrp			
< 60	(1)	–	–
60–69	1.058	0.963; 1.162	0.242
70–79	1.208	1.102; 1.323	< 0.0001
> 80	1.756	1.606; 1.921	< 0.0001
CCI			
0 (ref.)	(1)	–	–
1	1.447	1.355; 1.501	< 0.0001
2	1.339	1.243; 1.435	< 0.0001
> 2	1.857	1.735; 1.989	< 0.0001

During the study period, the mortality rate for females was reduced by 19% (*P* < 0.0001) compared with the rate for males. Compared with the age group < 60 years, the mortality rate was not different for the age group 60–79 (6% increase, *P* = 0.242), but was statistically significantly increased for the age groups 70–79 and > 80 years by 21% (*P* < 0.001) and 76% (*P* < 0.0001), respectively. The mortality rate decreased significantly over time by 6%, *P* < 0.000). Compared with no comorbidity (CCI = 0) the mortality rate increased with increasing comorbidity by 44, 34 and 86% (*P* < 0.0001 for all) for CCI = 1, CCI = 2, and CCI= > 2, respectively.

### Epidemiological projections 2016 through 2030

**Fig. 3:**
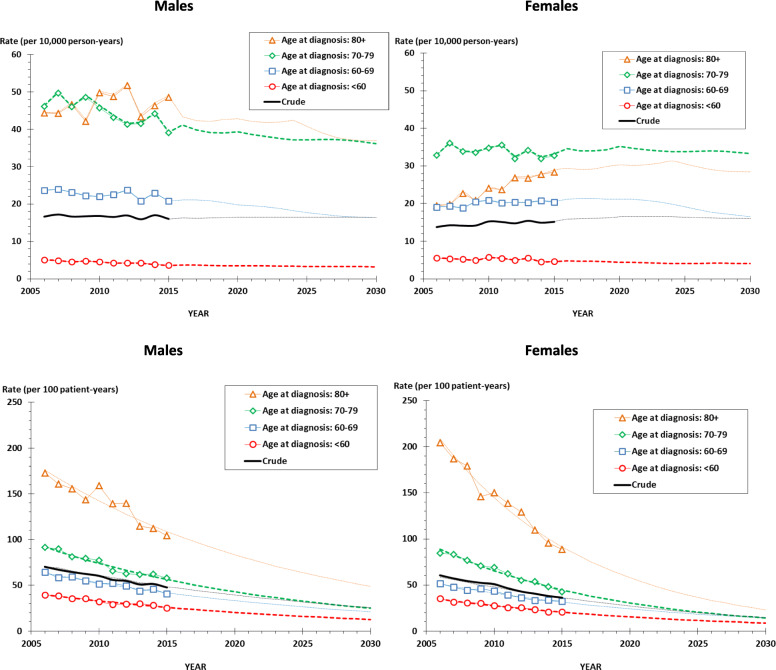
Incidence (top) and mortality (bottom) rates of lung cancer: Observed (full lines, with symbols) and projected (dotted lines) rates

The trends in the age-specific and crude summary incidence rates are shown for each sex separately in Fig. 3, upper panel. For males, all age-specific rates are assumed to decrease slightly during the projection period (2016 through 2030) according to the NORDCAN projections. However, the crude summary rate remains roughly constant reflecting that the Danish population is assumed to grow relatively more in the elderly age segments of higher lung cancer risk. For females, the pattern is more complex with an assumed further slightly increasing incidence rate for females aged 80+ years and constant or decreasing rates for the other age groups. The crude summary incidence rate for females will be marginally increasing. The mortality rate in lung cancer is assumed to decrease for both sexes and all age groups as well as for the crude summary rate according to the exponential projection model applied (Fig. 3, bottom panel). The assumed trends for the projection period fit well with the corresponding mortality rates obtained from the period with observations (Fig. 3, bottom panel).

**Fig. 4:**
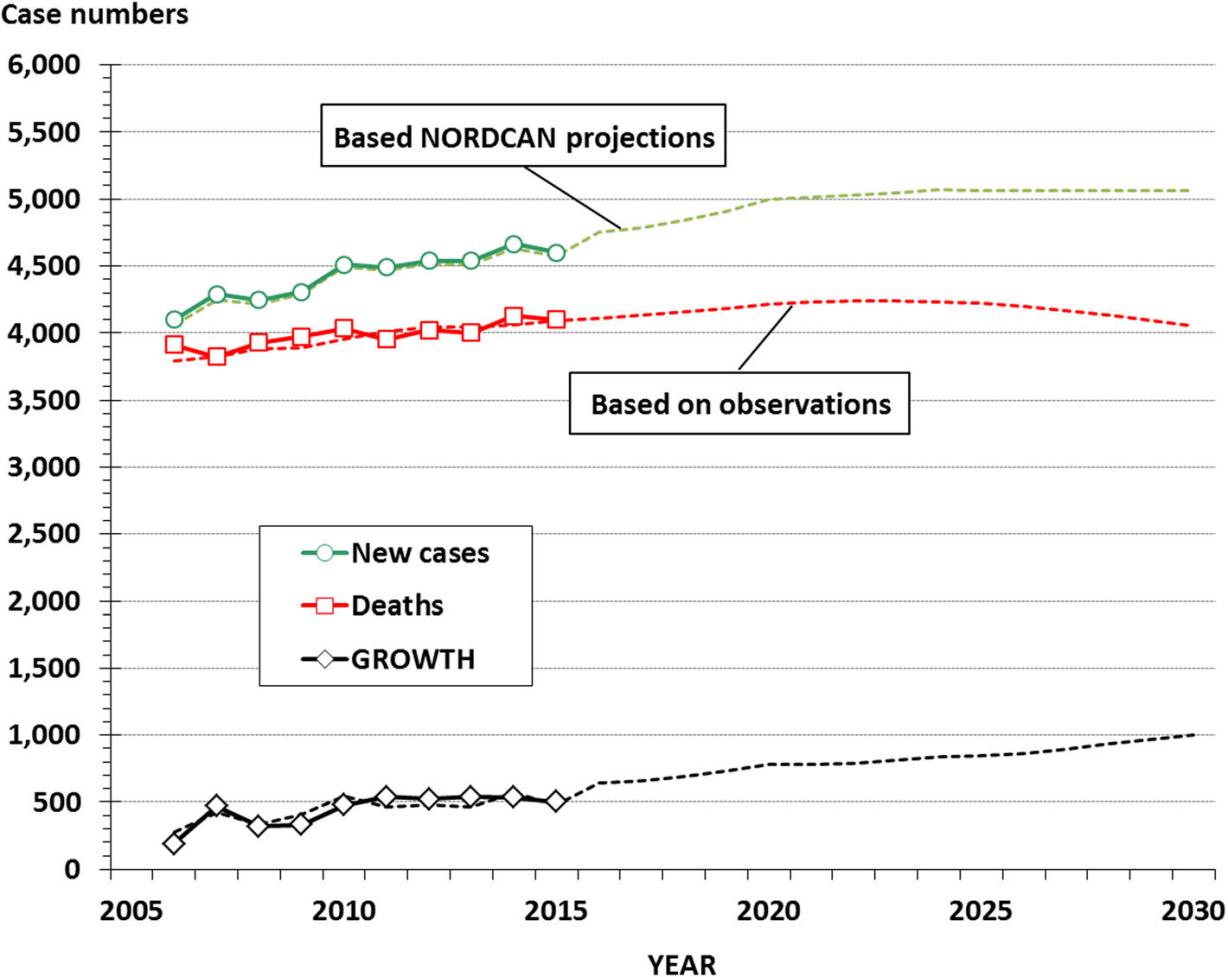
New cases and deaths in lung cancer: Observed (full lines, with symbols) and projected (dotted lines) case numbers, with estimated annual growth

Figure 4 shows the observed case numbers of incidence and mortality, with the corresponding annual growth (number of incident cases minus number of deaths). Also shown are the corresponding numbers projected by applying the projected sex and age-specific incidence and mortality rates (Fig. 3), combined with the projection of the demographical data for the population, to the flow and stock model. It is expected that the annual number of new cases of lung cancer will increase slightly until reaching a plateau around 2020. The annual number of deaths in the patient population is projected to increase modestly until about 2023 after which a slight decrease is expected. As a consequence, the annual growth in the patient population will grow continuously during the projection period.

**Fig. 5:**
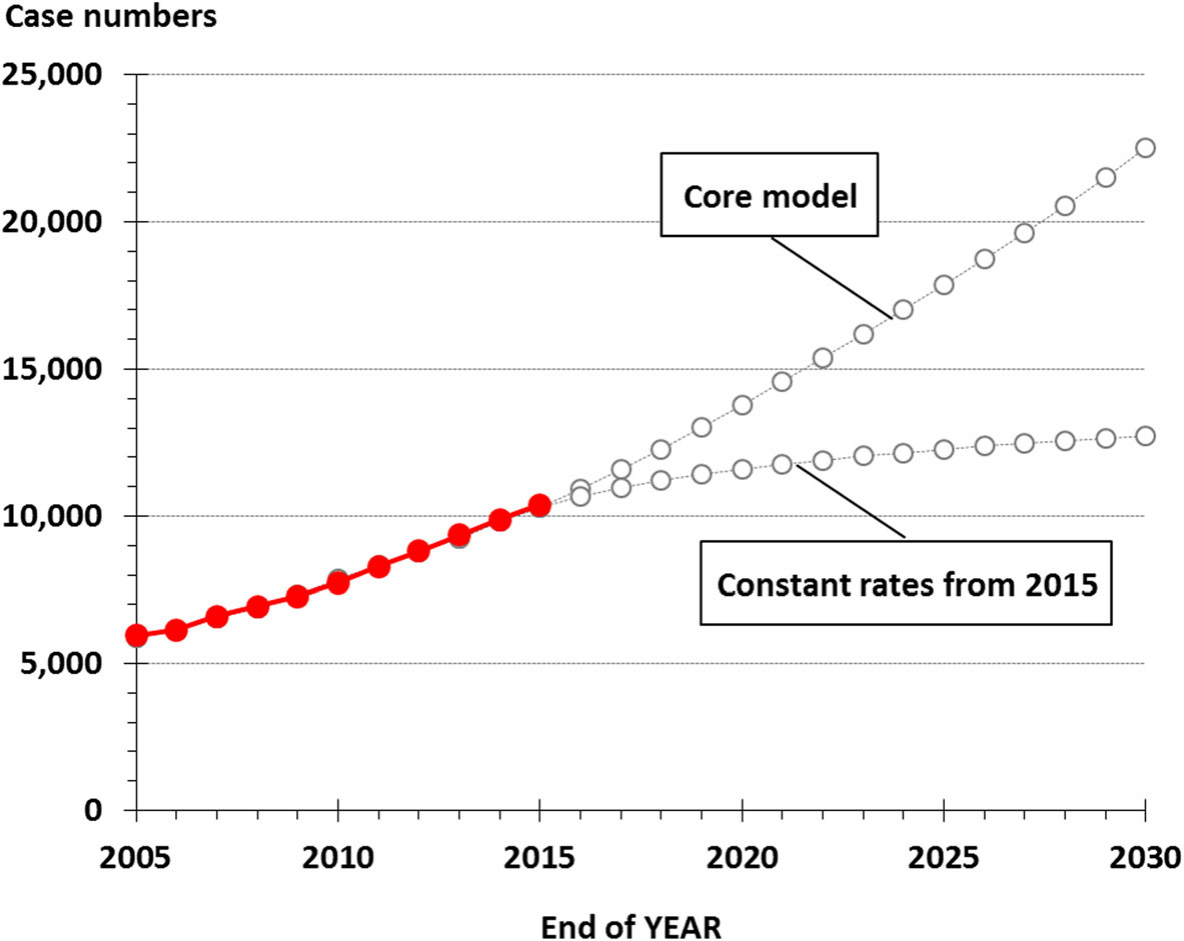
Observed and projected prevalence of LC: Observed (full lines, with filled symbols) and projected (dotted lines) number of prevalent cases

The trends in absolute case numbers of annual incidence and mortality (Fig. 4) are summarized for the prevalence in Fig. 5. In this model the numbers of prevalent cases of lung cancer in Denmark will more than double from about 10,000 at the end of 2015 to about 23,000 at the end of 2030. In the reference scenario with constant sex and age specific incidence and mortality rates, the increase in prevalence is less marked with an estimated number of prevalent cases at about 12,500 at the end of 2030.

## Discussion

This study reports an analysis of the clinical characteristics together with current and future epidemiological trends of lung cancer in Denmark.

Stage distribution of lung cancer has shifted towards lower stages at diagnosis. The background for the change is multifactorial and has to do with increased awareness of lung cancer in the population and easier access to advanced assessment such as CT scanning. Both with the effect that lung cancer is diagnosed in lower stages. In addition, more women with lung cancer and more adenocarcinomas were observed during the period, which are typically smaller and more peripheral than the previously dominant squamous cell type. Stages IV and IIIB that today primarily indicate oncological treatment with palliative intent and, optionally, in combination with targeted therapy or immune therapy, continue to be the dominant stage classifications albeit with a slight decrease from 2006 through 2015. At the same time, diagnosis in the stage classifications I to IIIA has increased from 25% in 2006 to 36% in 2015. The trend towards detecting lung cancer at an earlier stage provides a potential for offering curative treatment at an increasing rate, leading to improved prognosis. However, this trend is partly opposed by trends towards increasing age at diagnosis and a heavier load of comorbidity [14].

Apparently, the proportion of patients diagnosed with adenocarcinoma of the lung has increased markedly from 27% in 2006 to 45% in 2015. It is, however, questionable if this represents an actual change in the morphology pattern or whether the changes may be explained by increasing need for precise morphology classification and/or better diagnostic tools, including improved biopsy techniques. In the same period new guidelines on the classification of adenocarcinoma has been implemented worldwide and this probably has been of major impact for classifying large cell carcinomas and other primary lung cancers [15].

This analysis indicates that lung cancer in Denmark undergoes significant changes in the epidemiological profile. The incidence level is expected to decrease further, but the demographical changes will result in a modest increase in the annual absolute number of incident cases. In contrast, we expect a continuous reduction in the overall mortality rate, thereby increasing the annual growth in the prevalence to about 1000 in 2030 (Fig. 5).

Our results support that the epidemiology of lung cancer changes differently between the sexes. The incidence level is increasing for females in certain age groups, but the overall incidence level is still lower for females than for males. Yet, the background population at risk is growing relatively most for females in the higher age groups resulting in a trend towards an overweight of females among the new cases. This, combined with the substantially lower mortality in females than in males, results in a trend towards an increasing overweight of females in the prevalent population.

We estimate the prevalence of lung cancer in Denmark to more than double from the end of 2015 to the end of 2030. In the alternative scenario with constant incidence and mortality rates since 2015, the prevalence increase is much lower, due to the assumption of no further improvements in the prognosis of lung cancer. Since we have seen significant improvements in the prognosis for lung cancer in the most recent years and since we have not yet seen the expected effect of a number of improved treatments introduced in recent years (targeted therapy, immunotherapy and advanced radiotherapy) as well as an expected effect of early diagnosis we find it more likely with a continued improvement in the prognosis in lung cancer, the projections in the core model with the marked increasing prevalence is considered realistic.

Whereas the demographic evolution towards a relative increase in people of high age is a major driver of the increasing number of incident cases of lung cancer, our analysis demonstrates that the improvements in prognosis (as reflected in decreasing patient mortality rates) is by far the most important driver of the increasing prevalence of lung cancer.

The major strengths of this study are the validity of the data sources. The Cancer Registry, the Danish National Patient Register, and the Pathology Register are all believed to be complete and valid and as mentioned, the projected incidence and mortality rates follow the observed.

The limitations include the uncertainties associated with assumptions regarding the future levels of incidence and mortality. Changes in annual incident case numbers are driven by changes in the size and composition of the background population at risk of disease together with changes in the sex and age specific incidence rates. In addition to changes in demography and incidence rates, the changes over time in the annual number of prevalent cases are driven by changes in prognosis (here quantified as changes in the patient mortality rates) plus the effect of epidemiological disequilibrium, if present at the start of the projection period. Epidemiological disequilibrium exists if the annual number of new cases is not in balance with the number of deaths /regardless of cause) in the patient population. Under epidemiological disequilibrium, prevalence will change towards achieving a state of equilibrium. The model is well suited for changing assumptions, for example, implementation of screening programmes and new treatments.

Data on TNM and comorbidity are from the Cancer Registry and the Danish National Patient Register and are not validated by clinicians and they are associated with some degree of uncertainty. It is estimated that this uncertainty is of a minor magnitude and does not have a significant impact on the result. The effects of CCI must be interpreted with caution because resection status has not been included in the analysis and because of potential interaction with age. Finally, data on smoking habits have not been available for this study but the impact of changing smoking habits on the incidence rates of lung cancer observed so far will by means of NORDCAN’s projection methodology be reflected at least partly in the future incidence rates.

The worldwide lung cancer epidemic goes on. Years of focus on prevention and intervention against tobacco consumption has so far only had a modest effect on the incidence of lung cancer. Attempts over many years to develop more effective treatment methods have only marginally changed the prognosis of this deadly disease. In the light of the development of new treatment options and implementation of screening for lung cancer, a new optimism can be traced in the professional environment. Our data support that lung cancer is being diagnosed at an earlier stage, that incidence will stop increasing, and that mortality will decrease further. But these positive changes have implications in terms of a substantially increasing prevalence, with resources allocated to an increased demand for treatment, follow-up, and aftercare.

Projections of cancer incidence, mortality and prevalence are important for planning health services and to provide a baseline for assessing the impact of public health interventions. We present a dynamic, yet simple-spread sheet model to predict these changes and provide a comprehensive description of the current and future trends in the clinical characteristics and epidemiological profile of lung cancer. The model may be further developed by applying advanced statistical methods incl. age-period-cohort analysis of the raw data. We believe such modelling is an important tool in planning of lung cancer health care, particularly if updated periodically to account for the effects of preventive efforts and new treatment modalities. Realizing that forecasting models heavily depend on the assumed trends in incidence and mortality that drive the future prevalence, our simple and transparent tool makes it easy to establish alternative forecasting scenarios.

## Data Availability

Aggregated data and statistical programs may be made available upon request to corresponding author. Data are stored in a secure server environment at the host institution of the last author AG.

## References

[1] Jemal A, Bray F, Center MM (2011). Global cancer statistics. CA Cancer J Clin.

[2] Hovanec J, Siemiatycki J, Conway DI, Olsson A, Stücker I, Guida F, Jöckel KH, Pohlabeln H, Ahrens W, Brüske I, Wichmann HE, Gustavsson P, Consonni D, Merletti F, Richiardi L, Simonato L, Fortes C, Parent ME, McLaughlin J, Demers P, Landi MT, Caporaso N, Tardón A, Zaridze D, Szeszenia-Dabrowska N, Rudnai P, Lissowska J, Fabianova E, Field J, Dumitru RS, Bencko V, Foretova L, Janout V, Kromhout H, Vermeulen R, Boffetta P, Straif K, Schüz J, Kendzia B, Pesch B, Brüning T, Behrens T (2018). Lung cancer and socioeconomic status in a pooled analysis of case-control studies. PLoS One.

[3] Tataru D, Spencer K, Bates A, Wieczorek A, Jack RH, Peake MD, Lind MJ, Lüchtenborg M (2018). Cancer variation in geographical treatment intensity affects survival of non-small cell lung cancer patients in England. Cancer Epidemiol.

[4] Gjerstorff ML (2011). The Danish Cancer registry. Scand J Pub Health.

[5] Pedersen CB (2011). The Danish civil registration system. Scand J Pub Health.

[6] Schmidt M, Schmidt SAJ, Sandegaard JL, Ehrenstein V, Pedersen L, Sørensen HT (2015). The Danish National Patient Registry: a review of content, data quality, and research potential. Clin Epidemiol.

[7] Helweg-Larsen K (2011). The Danish register of causes of death. Scand J Pub Health.

[8] Bjerregaard B, Larsen OB (2011). The Danish pathology register. Scand J Pub Health.

[9] Danish Lung cancer Group (2016). Danish Lung cancer Registry Annual Report (in Danish).

[10] Goldstraw P, Crowley J, Chansky K, Giroux DJ, Groome PA, Rami-Porta R, Postmus PE, Rusch V, Sobin L, International Association for the Study of Lung Cancer International Staging Committee, Participating Institutions (2007). The IASLUNG CANCER lung cancer staging project: proposals for the revision of the TNM stage groupings in the forthcoming (seventh) edition of the TNM classification of malignant tumours. J Thorac Oncol.

[11] Quan H, Li B, Couris CM, Fushimi K, Graham P, Hider P, Januel J-M, Sundararajan V (2011). Updating and validating the Charlson comorbidity index and score for risk adjustment in hospital discharge abstracts using data from 6 countries. Am J Epidemiol.

[12] Green A, Sjølie AK, Eshøj O (1996). Trends in the epidemiology of IDDM during 1970-2020 in Fyn County, Denmark. Diabetes Care.

[13] Engholm G, Ferlay J, Christensen N, Bray F, Gjerstorff ML, Klint Å, Køtlum JE, Ólafsdóttir E, Pukkala E, Storm HH (2010). NORDCAN – a Nordic tool for cancer information, planning, quality control and research. Acta Oncol.

[14] Jakobsen E, Rasmussen TR, Green A (2016). Mortality and survival of lung cancer in Denmark: Results from the Danish Lung cancer Group 2000–2012. Acta Oncol.

[15] Travis WD, Brambilla E, Noguchi M, Nicholson AG, Geisinger KR, Yatabe Y, Beer DG, Powell CA, Riely GJ, van Schil PE, Garg K, Austin JHM, Asamura H, Rusch VW, Hirsch FR, Scagliotti G, Mitsudomi T, Huber RM, Ishikawa Y, Jett J, Sanchez-Cespedes M, Sculier JP, Takahashi T, Tsuboi M, Vansteenkiste J, Wistuba I, Yang PC, Aberle D, Brambilla C, Flieder D, Franklin W, Gazdar A, Gould M, Hasleton P, Henderson D, Johnson B, Johnson D, Kerr K, Kuriyama K, Lee JS, Miller VA, Petersen I, Roggli V, Rosell R, Saijo N, Thunnissen E, Tsao M, Yankelewitz D (2011). International association for the study of lung cancer/american thoracic society/european respiratory society international multidisciplinary classification of lung adenocarcinoma. J Thorac Oncol.

